# Determining the Relationship Between Hot Flushes and LH Pulses in Menopausal Women Using Mathematical Modeling

**DOI:** 10.1210/jc.2018-02797

**Published:** 2019-04-15

**Authors:** Julia K Prague, Margaritis Voliotis, Sophie Clarke, Alexander N Comninos, Ali Abbara, Channa N Jayasena, Rachel E Roberts, Lisa Yang, Johannes D Veldhuis, Krasimira Tsaneva-Atanasova, Craig A McArdle, Waljit S Dhillo

**Affiliations:** 1 Section of Endocrinology & Investigative Medicine, Imperial College, London, United Kingdom; 2 College of Engineering, Mathematics, and Physical Sciences, University of Exeter, Exeter, United Kingdom; 3 EPSRC Centre for Predictive Modelling in Healthcare, University of Exeter, Exeter, United Kingdom; 4 Department of Endocrinology, Imperial College Healthcare NHS Trust, London, United Kingdom; 5 Mayo Clinic, Rochester, Minnesota; 6 Living Systems Institute, University of Exeter, Exeter, United Kingdom; 7 Bristol Medical School, University of Bristol, Bristol, United Kingdom

## Abstract

**Background:**

Hypothalamic kisspeptin/neurokinin B/dynorphin (KNDy) neurones regulate LH pulsatility. It is widely accepted that the menopausal hot flush (HF) consistently synchronizes with the LH pulse, implicating the hypothalamic KNDy neurones in generating LH pulsatility and HF. Using a modern immunoassay and mathematical modeling, we investigated if the HF and LH pulse were consistently synchronized in menopausal women.

**Methods:**

Eleven menopausal women (51 to 62 years of age and experiencing ≥7 HF in 24 hours) participated in an 8-hour study. Subjects self-reported HF and underwent peripheral blood sampling every 10 minutes. LH pulsatility was determined using two mathematical models: blinded deconvolution analysis and Bayesian spectrum analysis. The probability that the LH pulse and HF event intervals matched was estimated using the interval distributions observed in our data.

**Results:**

Ninety-six HFs were self-reported, and 82 LH pulses were identified by blinded deconvolution analysis. Using both models, the probability that the two event intervals matched was low in the majority of participants (mean *P* = 0.24; *P* = 1 reflects perfect association).

**Interpretation:**

Our data challenge the widely accepted dogma that HFs consistently synchronize with an LH pulse and therefore have clinically important therapeutic and mechanistic implications.

## Teaser

Using mathematical modeling to determine the relationship between hot flushes and LH pulses in menopausal women challenges the long-held dogma that the menopausal flush synchronizes with the LH pulse.

Seventy percent of women experience hot flushes (HFs) secondary to the decline in circulating estrogen levels associated with the menopause (1), and 10% describe them as intolerable (2). Symptoms are typically long-lasting (median, 7.4 years) (3) and disrupt all aspects of daily life. Their precise etiology has been of considerable interest to the scientific community for many years with many differing explanations suggested, including a narrowed thermoneutral zone (4), altered central concentrations of neurotransmitters including serotonin and noradrenaline (5), and modulation of the thermoregulatory pathway (6, 7). Some hypotheses have been tested with the clinical application of an associated therapeutic or intervention with some achieved benefit (*e.g.*, selective serotonin reuptake inhibitors to alter central concentrations of the neurotransmitter serotonin) (8). However, since the publication of two seminal papers in 1979, it has been repeatedly referenced and accepted that the menopausal flush synchronizes with the onset of the LH pulse (9, 10). In these two separate papers involving 12 menopausal women experiencing frequent HFs, skin temperature was measured using a finger probe together with regular sampling of peripheral blood for LH, and participants self-reported onset of HFs for at least an 8-hour study period (9, 10). Tataryn *et al.* (10) defined LH pulses as increases in LH of 20% over nadir as per Santen and Bardin criteria (11); no such methodological description was outlined by Casper *et al.* (9). Casper *et al.* (9) concluded that the reported HFs occurred in direct association with the onset of the LH pulse in all instances. In the Tataryn cohort, nearly all measured skin temperature increases were reported to be associated with a reported flush (32/34), and 26 of the 31 LH pulses measured had “a close temporal relationship to the temperature elevations,” although how this was determined is not described in detail (10).

Since publication of these seminal papers, LH immunoassays have improved, sophisticated methods for pulse analysis have been developed, and uncoupling of LH pulses from HFs has been demonstrated in a number of clinical settings, including hypothalamic amenorrhea and hypopituitarism, which are discussed in detail herein (12). We further explored the hypothesis that LH peak synchronizes with HFs in menopausal women to determine the relationship between HFs and LH because this could contribute to further mechanistic understanding and/or suggest therapeutic targets, such as the ongoing development of neurokinin B (NKB) antagonists, as treatments for HFs (13).

To achieve fine control within a biological system, hormones tend to be secreted in a combination of two patterns: a continuous pattern or a background (basal) pattern with interspersed acute patterns of sudden and transient bursts (pulses or peaks). The relationship between these patterns of secretion varies between species, age, and context, including disease state (14). One approach to interpreting such patterns of hormone secretion over a monitored time period is to review the graphs of hormone concentration measured and to mark the basal rate and peaks by eye. However, this is overly simplistic because it does not account for elimination rates of the measured hormone (clearance), half-life, or whether predefined numerical criteria should be applied to distinguish a true peak from random assay variability (e.g., a sudden rise that was two or three times the assay coefficient of variation) (14). Such complex regulatory mechanisms require greater detail and flexibility in the method used to interpret secretion patterns. Mathematical modeling has been key in achieving this because it can begin to address the random effects that arise, such as procedural inconsistences (missing data, outliers) and measurement variability, host factors, and biological variability due to the inconsistent or variable pulse-to-pulse drive (14).

One such mathematical approach is that of blinded deconvolution analysis, which can incorporate multiple analytical algorithms with independent assumptions to reliably determine valid estimates of underlying basal and pulsatile secretion and/or elimination rates from a hormone concentration profile (14). This approach has been particularly useful in analyzing LH pulse dynamics and is now considered a robust and established methodology (14). Furthermore, including the approximate entropy statistic quantifies the relative consistency of patterns in sequential measurements within the hormone concentration profile (*i.e.*, it incorporates a measure of how ordered the pattern of hormone secretion is, where zero denotes perfect orderliness) and in doing so accounts for some of the important physiological variation in pulsatile secretion (*e.g.*, the strikingly different pattern regularity in LH secretion in postmenopausal compared with premenopausal women).

Bayesian methods have increasingly been used across varied disciplines to quantify uncertainty among measurements and hypotheses (15). In doing so, Bayes theorem can be adopted to apply mathematical rules to subjective probabilities that are influenced by an individual’s degree of belief that an event will occur and their state of knowledge regarding the event at the time to generate an estimate of certainty from a probability distribution for an observation (15). This approach can be robustly applied to assessing hormone secretion patterns and more specifically to estimating their pulse intervals along with the associated uncertainty.

In this study, we determined whether the LH peak was associated with HFs in menopausal women using a modern LH immunoassay and two independent and established mathematical models. Blinded deconvolution analysis was used to identify LH peaks and the likelihood that they coincide with self-reported HFs, and Bayesian spectrum analysis was used to determine the probability distribution for LH pulse intervals and the probability of a match between the LH peak and self-reported HF intervals.

## Patients and Methods

### Protocol

Eleven participants attended our temperature-controlled clinical research facility for a single 8-hour study visit. Once a participant was introduced to the unit, a cannula was inserted in a peripheral vein under aseptic conditions (time −30 minutes), through which all blood samples were taken every 10 minutes from time 0 until time 480 minutes. All participants were ambulatory and could eat and drink freely during the study visit. Precise timings of self-reported HF episodes were recorded in real time. All blood samples were left to clot for at least 30 minutes prior to centrifugation at 503rcf for 10 minutes, after which the serum supernatant was extracted and immediately frozen at −20°C for analysis using an automated chemiluminescent immunoassay method (Abbott Diagnostics, Maidenhead, UK) in batches after study completion. Reference ranges were as follows: LH, 4 to 14 IU/L; respective intra-assay and interassay coefficients of variation, 4.1% and 2.7%; analytical sensitivity, 0.5 IU/L.

### Statistical analysis

LH pulsatility was determined from the raw data of LH measurements over the 8-hour study period using two independent mathematical models. Using a blinded deconvolution (empirical) method with 93% sensitivity and 93% specificity, we calculated onset times and pattern regularity of the LH pulses (orderliness) as per Veldhuis *et al.* (14). Using Bayesian spectrum analysis (BSA), we calculated the probability density distribution for interpulse interval for each participant conditional on the observed data. The BSA method was then applied to the accompanying self-reported HF event data to obtain the posterior distribution for the interval between episodes. To achieve this, HF event data were transformed into a binary (0-1) time series, where 1 indicates an HF episode and 0 indicates no episode. BSA was performed in R using the BaSaR library (Bayesian Spectrum Analysis in R) (16).

For each participant, the two distributions of LH pulse and HF intervals were used to investigate the association between HFs and LH pulses. A perfect association between LH pulses and HF events requires that the corresponding intervals are equal. Therefore, it is possible to use the two distributions to calculate the probability that the intervals match (*i.e.*, are “approximately” equal to each other) and use this probability to denote the degree of correlation between the occurrence of LH pulses and HF events. This approach is illustrated for simulated (*i.e.*, artificially created or synthetic) data in the online repository (17), where the probability was calculated using the following formula:

$$P_{\text{match}}=\overset{T_{\text{max}}}{\underset{T_{\text{min}}}{\int }}P_{\text{LH}}\left(t|D\right)\left(\overset{t+e/2}{\underset{t-e/2}{\int }}P_{\text{HF}}\left(t^{\prime }|D\right)dt^{\prime }\right)dt$$

where *P*_LH_(*t*/*D*) and *P*_HF_(*t*′|*D*) are the posterior distributions for *T*_LH_ (LH pulse intervals) and *T*_HF_ (HF intervals); *T*_min_ = 20 minutes and *T*_max_ = 240 minutes specify the range for *T*_LH_ and *T*_HF_; and the parameter *e* determines the acceptable discrepancy between the intervals (*i.e.*, intervals match if |*T*_LH_ − *T*_HF_| ≤ *e*/2). We estimated the probability for *e* = 10 minutes, which is the LH sampling interval in our clinical study, and for *e* = 20 minutes, which is twice the LH sampling interval. This allowed us to test the robustness of our method against variations of this parameter.

**Figure 1.**
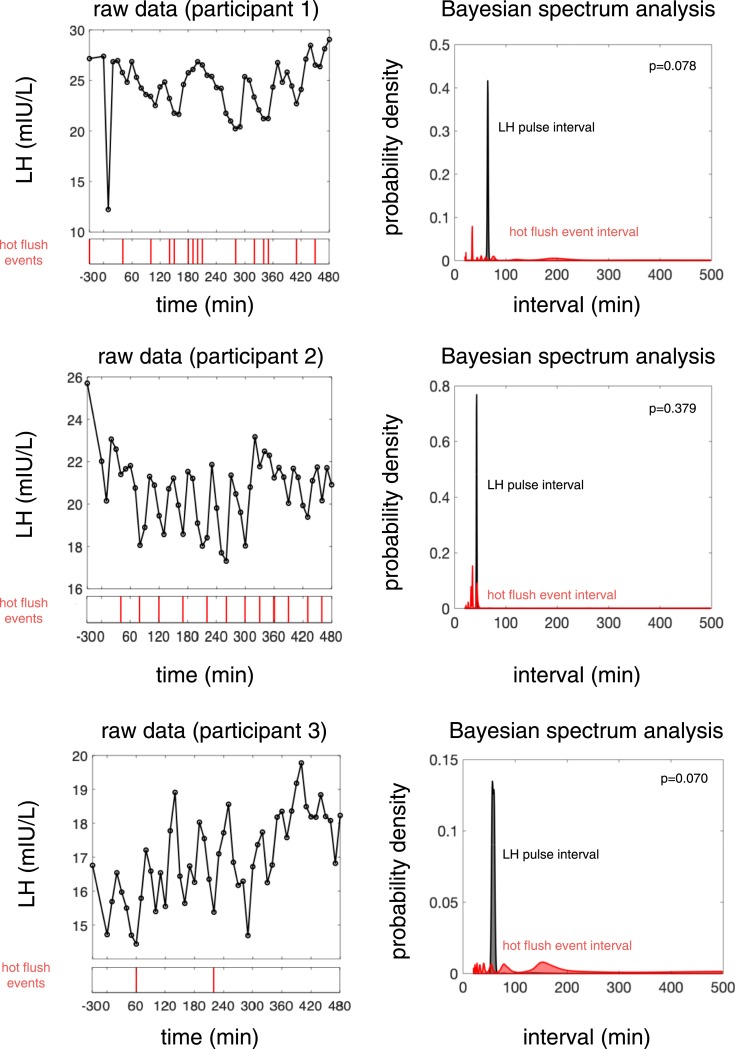

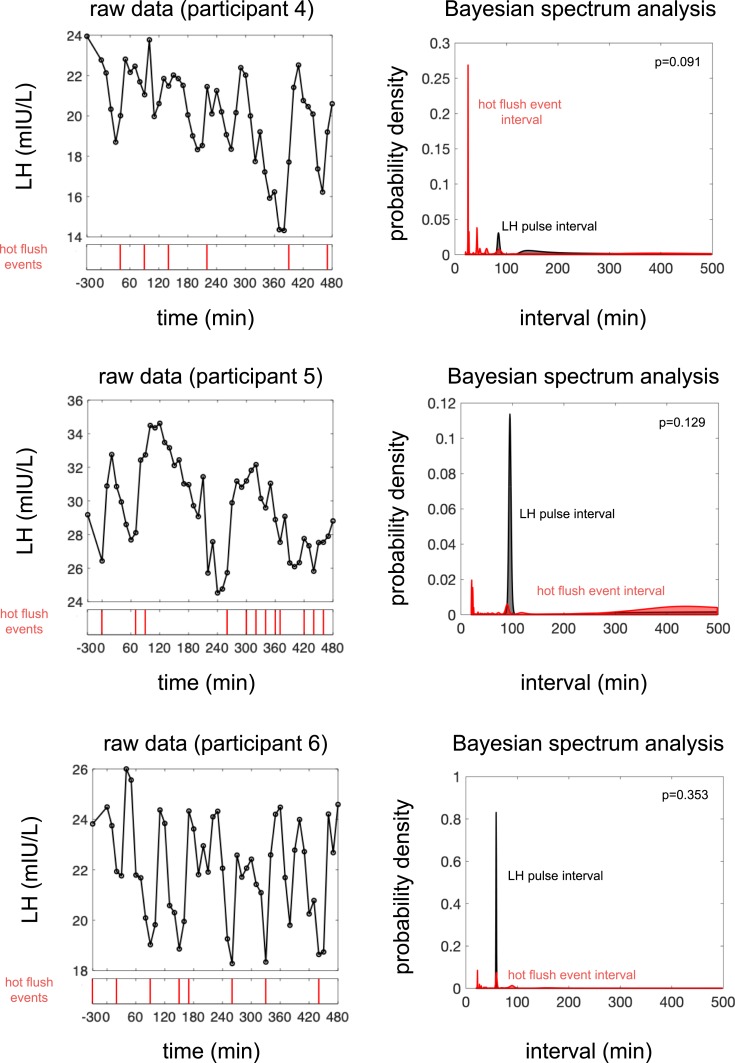

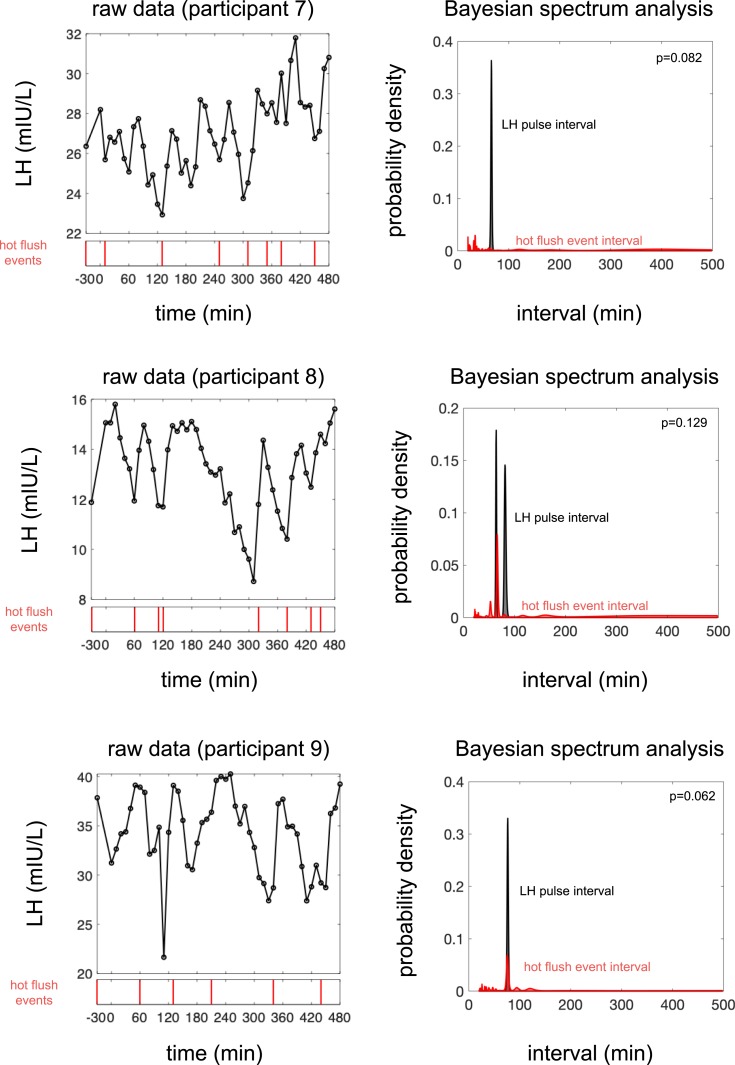

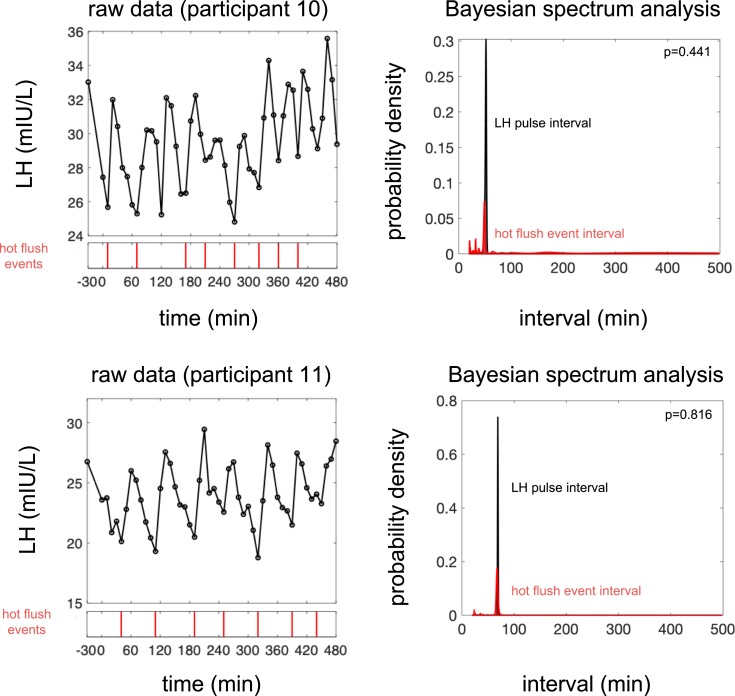
Individual raw data for all participants and the corresponding histograms for LH pulses and HF event intervals as determined by BSA. For each participant: (Left panel) Black line: LH (mIU/L) measured every 10 min from a peripheral indwelling venous cannula; red line: self-report of an HF. (Right panel) Posterior distribution of LH pulse interval (black) and HF event interval (red) as determined by BSA. The probability of the two intervals (LH pulse and HF) matching is calculated, where *P* = 1 reflects a perfect association.

To further supplement our analysis, we estimated the probability that the LH pulse and HF event intervals match (*i.e.,* are equal) using the empirical distributions of intervals observed in our data. To do so, for each participant we calculated (i) the intervals between self-reported HF events and (ii) the intervals between the onset of LH pulses that were identified by the blinded deconvolution method (14). Then, we calculated the probability that the LH pulse and HF intervals match as the fraction of pairs from the two interval groups (LH pulse and HF) that are matched within *e* minutes, where, similar to the Bayesian analysis, *e* was set to 10 minutes and 20 minutes. Ten minutes was selected because this was the frequency of blood sampling, and 20 minutes was selected because this was double the length of the frequency of blood sampling.

### Study approval

Ethical approval was obtained from the West London Regional Ethics Committees (15/LO/1481). All participants provided written informed consent prior to inclusion. The study was performed in accordance with Good Clinical Practice guidelines. Eligible participants were healthy women aged 40 to 62 years, experiencing at least seven HFs per 24-hour period (some of which were bothersome or severe) who had not had a menstrual period for at least 12 months and who had not been taking any medication shown to improve menopausal flushes in the preceding 8 weeks.

## Results

The cohort of 11 participants had a mean age of 56 years (range, 51 to 62 years) and a mean body mass index of 26.0 kg/m^2^ (range, 19.7 to 36.7 kg/m^2^). Eight of the participants were white, and three were Black Caribbean. The mean time since onset of HFs was 103 months (range, 20 to 192 months), and the mean time since last menstrual period was 104 months (range, 36 to 192 months). Three subjects were current smokers, and mean LH at screening was 28 IU/L (SD, 7.2). All possible covariates were included in exploratory analyses of LH pulse number, amplitude, and orderliness as per our *a priori* statistical plan but were removed from the final analysis models when not shown to be significant.

The total number of HFs reported was 96, and the total number of LH pulses identified by deconvolution analysis was 82. The mean number of HFs per participant was nine (SD, 4.38; range, 2 to 19), and the mean number of LH pulses per participant was eight (SD, 1.97; range, 4 to 10) (Table 1). For both methods (BSA and blinded deconvolution analysis), the probability of a match for the intervals between the LH pulses and HFs varied greatly between participants (lowest *P* = 0.062 to highest *P* = 0.816, where *P* = 1 reflects a perfect match) (Table 1). For the majority of participants (8/11), the probability of a match was <0.5 (irrespective of the method used and of whether intervals were matched within 10 or 20 minutes), suggesting a weak association between the occurrence of the HF and the LH pulse in the majority of menopausal women (Table 1).

**Table 1.** Summary of Results

Participant	Number of LH Pulses	Number of HFs	LH Pulse Interval	HF Interval	Probability Match Within 10 Min (BSA)	Probability Match Within 10 Min (Empirical)	Probability Match Within 20 Min (BSA)	Probability Match Within 20 Min (Empirical)
1	7	19	75.00	34.29	0.078	0.190	0.129	0.298
2	4	12	113.3	38.18	0.379	0.333	0.355	0.333
3	9	2	52.50	160.00	0.070	0.000	0.173	0.000
4	7	6	71.67	86.00	0.091	0.333	0.163	0.467
5	6	12	92.00	41.82	0.129	0.218	0.199	0.364
6	10	8	55.56	67.14	0.353	0.429	0.240	0.556
7	5	8	80.00	68.57	0.082	0.250	0.151	0.357
8	10	8	54.44	68.57	0.129	0.254	0.369	0.397
9	8	6	62.86	94.00	0.062	0.200	0.104	0.343
10	9	8	55.00	55.71	0.441	0.518	0.514	0.679
11	7	7	66.67	66.67	0.816	0.806	0.887	0.972

Outcomes and probabilities that the intervals between LH pulses and HF episodes match within 10 and 20 min as determined by BSA and an empirical method (blinded deconvolution analysis) as per Veldhuis *et al.* (14). A probability match of 1 reflects a perfect association between timing of LH pulses and menopausal HFs.

The individual participant histograms for HF and LH pulse interval enable an estimate of certainty to be deduced from the shape of the distribution (*i.e.*, narrow distribution suggests increased certainty; wide distribution with multiple peaks suggests low certainty), which reflects the extent of the variation in the certainty of the frequency estimate for both intervals between participants. This is illustrated for simulated (*i.e.*, artificially created or synthetic) data in the online repository (17). Figure 1 incorporates all raw data for all 11 menopausal women in our study.

**Figure 2.**
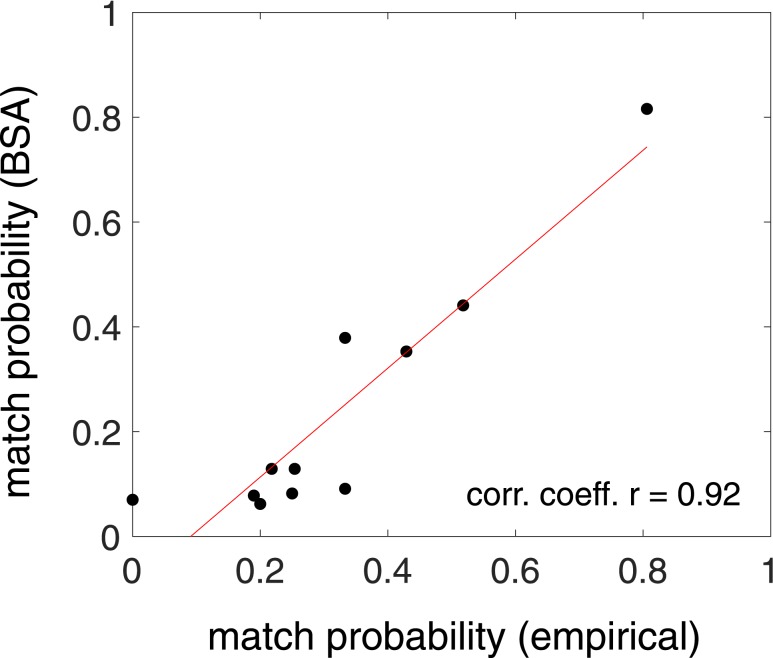
Summary plot of the probability that the intervals between LH pulses and HF episodes are matched within 10 min as determined by BSA (*y*-axis) and an empirical method (blinded deconvolution analysis; *x*-axis) for all 11 participants (each marked by a black circle). Correlation coefficient r = 0.92 (r = 1 reflects a perfect linear association), suggesting good concordance between the two mathematical models.

Good concordance was demonstrated between the BSA and empirical method in calculating the probability that the intervals between LH pulses and HF episodes matched (Fig. 2), although the match probabilities were typically a little higher when intervals where matched within 20 minutes rather than within 10 minutes.

## Discussion

Using two independent and established mathematical models, we have demonstrated that, in the majority of menopausal women in this study, there was no clear association between LH pulse and HF interval (Table 1). This is in contrast to the repeatedly referenced and accepted long-held conclusion that the LH pulse synchronizes with the onset of the menopausal HF. In only one menopausal woman out of 11 did we determine a high probability (*P* > 0.8) that the intervals between LH pulse and HF episodes matched. Furthermore, in contrast to Casper *et al.* (9), we did not find that the women who had the most frequent LH pulses had the highest frequency of HF episodes. Because the methodological detail regarding how LH pulses were defined is not included in the paper by Casper *et al.* (9), further interpretation or inference as to why these two analyses have conflicting results is problematic. Furthermore, using improved methodology (modern LH assay and robust mathematical analysis of LH pulsatility), we did not confirm the close temporal association between LH pulse and HF episode reported in the paper by Tataryn *et al.* (10), where LH pulses were defined as increases of LH of 20% over nadir.

However, the presence of HFs in other clinical conditions that affect the secretion of LH from the pituitary and/or GnRH neurones suggests that, in some circumstances, the synchronicity between LH secretion and HFs suggested by Casper *et al.* (9) and Tataryn *et al.* (10) is not seen. For example, women with hypopituitarism still experience HFs if administered exogenous estrogen that is subsequently withdrawn despite having no ability to secrete LH (12). Similarly, premenopausal women who are administered GnRH agonists to suppress secretion of LH and sex steroids for a condition such as endometriosis also experience HFs on treatment (18). However, whereas LH pulses are due to the pulsatile secretion of GnRH (19), GnRH secretion itself cannot be the sole causative factor of HFs because exogenous estrogen withdrawal in female patients with a genetic cause of hypogonadotrophic hypogonadism secondary to failure of the hypothalamic GnRH neurones to develop and migrate appropriately (Kallman syndrome) causes HFs (20). Furthermore, HFs do not occur in hypothalamic amenorrhea where estrogen and GnRH levels are both low in response to a physiological stress such as undernutrition (20).

The recent discovery that highly conserved hypothalamic kisspeptin/neurokinin B/dynorphin (KNDy) neurones that colocalize kisspeptin, NKB, and dynorphin act upstream of the GnRH neurones to regulate the secretion of LH from the anterior pituitary has changed the understanding of the neuroendocrine control of reproduction (21, 22). Within this context, our data indicate two possible scenarios. The first scenario is that the pulsatile kisspeptin output from KNDy neurones that drives pulsatile GnRH and LH secretion also drives the HFs, in which case we would have to invoke an additional regulator of the HFs to explain the uncoupling. The second and more likely scenario is that the HFs are driven by a separate, independent output from the KNDy neurones so that the HFs are not necessarily synchronized with kisspeptin, GnRH, or LH pulses. NKB is the obvious possibility for the alternative output because it has a well-established role in the reproductive and thermoregulatory neuronal pathways (23) and therefore has a role in the etiology of HFs secondary to sex-steroid deficiency, such as menopause (23–26), but does not significantly contribute to the regulation of pulsatile LH secretion. If this were the case, it might be possible to consider LH pulses and HFs as readouts for such distinct KNDy neurone outputs and to manipulate them independently in therapeutic settings. Although speculative, this possibility warrants further investigation. This could be achieved either using mathematical modeling and/or other basic science experiments to offer possible mechanisms to explain the variation in the extent of association between LH pulse and HF interval between individuals, including the possibility of genetic variation in the neurokinin 3 receptor gene (*TACR3*), as suggested by Crandall *et al.* (25).

Our data were collected in real-time from a representative cohort of participants with varied body mass index, and ethnic background, and smoking status who were ambulatory and eating freely within a temperature-controlled environment and undergoing regular venous sampling, and therefore our data should be reliable. The data have been analyzed using two independent and established mathematical models that have different limitations and yet have shown good concordance, suggesting that the results are reliable. We acknowledge a limitation of the current Bayesian spectrum analysis is that the model assumes fixed LH pulse intervals, and we know that physiologically this is not the case.

In summary, using a modern LH immunoassay and two independent, established mathematical models, we have demonstrated that LH pulses and HFs were not consistently synchronized in the majority of menopausal women in this study. It is well established that these events can be uncoupled in conditions such as hypothalamic amenorrhea or estradiol withdrawal from women with hypopituitarism, but our data clearly suggest that such uncoupling is also highly prevalent in menopausal women. The clear implication is that other factors contribute to their etiology, raising the future possibility of more specific therapeutic approaches, such as NKB antagonists, for menopausal HFs.

## Abbreviations:

BSA: Bayesian spectrum analysis
HF: hot flush
KNDy: kisspeptin/neurokinin B/dynorphin
NKB: neurokinin B
